# The Role of Physical Exercise and Rehabilitative Implications in the Process of Nerve Repair in Peripheral Neuropathies: A Systematic Review

**DOI:** 10.3390/diagnostics13030364

**Published:** 2023-01-18

**Authors:** Rita Chiaramonte, Vito Pavone, Gianluca Testa, Isabella Pesce, Dalila Scaturro, Giuseppe Musumeci, Giulia Letizia Mauro, Michele Vecchio

**Affiliations:** 1 Section of Pharmacology, Department of Biomedical and Biotechnological Sciences, University of Catania, 95124 Catania, Italy; 2 Section of Orthopaedics and Traumatology, Department of General Surgery and Medical Surgical Specialties, University Hospital Policlinico “Rodolico-San Marco”, University of Catania, 95123 Catania, Italy; 3 Department of Surgery, Oncology, and Stomatology, University of Palermo, Via Liborio Giuffrè 5, 90127 Palermo, Italy; 4 Department of Biomedical and Biotechnological Sciences, Anatomy, Histology and Movement Sciences Section, University of Catania, 95124 Catania, Italy; 5 Rehabilitation Unit, “AOU Policlinico Vittorio Emanuele”, 95124 Catania, Italy

**Keywords:** peripheral neuropathy, physical activity, exercise, nerve regeneration, humans, animals, nerve stimulation

## Abstract

Background: The various mechanisms involved in peripheral nerve regeneration, induced by exercise and electrical nerve stimulation, are still unclear. Objective: The aim of this review was to summarize the influence of physical exercise and/or electrical stimulation on peripheral nerve repair and regeneration and the variation of impact of intervention depending on timing, as well as kind and dosage of the intervention. A literature survey was conducted on PubMed, Scopus, and Web of Science, between February 2021 to July 2021, with an update in September 2022. Methodology: The literature search identified 101,386 articles with the keywords: “peripheral nerve” OR “neuropathy” AND “sprouting” OR “neuroapraxia” OR “axonotmesis” OR “neurotmesis” OR “muscle denervation” OR “denervated muscle” AND “rehabilitation” OR “physical activity” OR “physical exercise” OR “activity” OR “electrical stimulation”. A total of 60 publications were included. Eligible studies were focused on evaluating the process of nerve repair (biopsy, electromyographic parameters or biomarker outcomes) after electrical stimulation or physical exercise interventions on humans or animals with peripheral sensory or motor nerve injury. Synthesis: This study shows that the literature, especially regarding preclinical research, is mainly in agreement that an early physical program with active exercise and/or electrical stimulation promotes axonal regenerative responses and prevents maladaptive response. This was evaluated by means of changes in electrophysiological recordings of CMAPs for latency amplitude, and the sciatic functional index (SFI). Furthermore, this type of activity can cause an increase in weight and in muscle fiber diameter. Nevertheless, some detrimental effects of exercising and electrical stimulation too early after nerve repair were recorded. Conclusion: In most preclinical studies, peripheral neuropathy function was associated with improvements after physical exercise and electrical stimulation. For humans, too little research has been conducted on this topic to reach a complete conclusion. This research supports the need for future studies to test the validity of a possible rehabilitation treatment in humans in cases of peripheral neuropathy to help nerve sprouting.

## 1. Introduction

Peripheral neuropathy can result from damage to and dysfunctions of motor, sensory or autonomic peripheral nerves. Moreover, denervation and disuse lead to the loss of muscle mass [1]. Thus, peripheral neuropathies are highly disabling, negatively affecting mobility and reducing autonomy in the activities of daily living [2].

No specific rehabilitative therapies have been accepted as a standardized treatment for peripheral neuropathy-related symptoms [3]. For this reason, research in this field is very interesting. For example, treadmill training seems to promote locomotor recovery in mouse models with chronic spinal cord injury, influencing neurotrophin expression such as Brain-Derived Neurotrophic Factor (BDNF) [4]. Furthermore, therapeutic exercise and rehabilitative strategies have been proposed to influence axon regeneration in the peripheral nervous system [5] and prevent peripheral nerve dysfunction in animals [6,7]. Numerous types of physical exercise, such as passive range-of-motion exercises, active-assisted exercises, locomotion (gait, treadmill and running training), and electrostimulation (a direct stimulation of the muscle fiber with an electric charge) of denervated muscle, have been proposed as effective strategies for retarding muscle atrophy and improving contractility after reinnervation [6,8,9,10,11,12]. Moreover, these strategies seem to upregulate genes and kinases associated with neuronal plasticity in the spinal cord and skeletal muscle [13], such as the BDNF [7,10,13,14,15,16,17], especially in the presence of the glia maturation factor (GMF) [18], calcitonin gene-related peptide (CGRP) related axonal regeneration [19,20], GAP43 [16,21], and phospho-ERK1/2 protein [21] involved in the recovery of peripheral nerve injury.

Electrical stimulation is a type of physical therapy treatment used to accomplish various tasks in physical therapy. According to research on transplanted nerves, it accelerates the regeneration rate of reconstructed peripheral nerves [22,23]. For this reason, it is interesting to assess the response during the process of nerve repair in peripheral neuropathies.

The paucity of literature on the relationship between peripheral neuropathy biomarkers and physical exercise in humans makes it necessary to extend our research to animal studies to better understand the mechanisms of nerve regeneration related to exercise. The aim of this systematic review was to determine whether physical exercise and electrical stimulation play a role in peripheral nerve repair and regeneration, and especially to understand the mechanisms by which electrical stimulation and physical exercise influence recovery after peripheral neuropathies. Furthermore, the aim was to highlight the impact of these specific strategies, their timing, kind, and dosage, especially regarding when to start this treatment, immediately after injury or during nerve regeneration, among humans and animals with peripheral neuropathy or nerve injury.

## 2. Materials and Methods

### 2.1. Information Sources and Database Search

The search was conducted on the following medical electronic databases: PubMed, Scopus, and Web of Science. The reference list of related articles was also used to identify any other suitable documents. The search strategy was conducted from February 2021 to July 2021, with an update in September 2022. The search used the following terms and keywords: “peripheral nerve” OR “neuropathy” AND “sprouting” OR “neuroapraxia” OR “axonotmesis” OR “neurotmesis” OR “muscle denervation” OR “denervated muscle” AND “rehabilitation” OR “physical activity” OR “physical exercise” OR “activity” OR “electrical stimulation”.

The review protocol was registered in the PROSPERO database of systematic reviews (www.crd.york.ac.uk/Prospero with the registration number CRD42021248509, accessed on 8 July 2021).

### 2.2. Eligibility Criteria

Studies were included in this systematic review when they described the mechanisms linked to electrical stimulation and physical exercise that influenced the process of nerve repair in peripheral neuropathies. Moreover, the studies were included according to the Population, Intervention, Comparison, Outcome, and Study Design (PICOS) criteria that the authors followed: the participants were animals (rats, rabbits, snails) and adult humans with sensory and/or motor peripheral nerve injuries, or neuropathies or muscle denervation; intervention was based on physical exercises, electrical stimulation, rehabilitation therapy, and forced immobility; the comparators included hypomobility/immobility, or absence of nerve injury; the outcomes were represented by changes in electrophysiological values, muscle and axon characteristics (muscle weight, muscle fibers and axon diameter), biomarkers and sciatic functional index (SFI); study design was preclinical and clinical experimental studies. Articles about chemotherapy-induced peripheral neuropathy were not added because the particularity of the topic and the necessity of proper research about the mechanisms of nerve repair after neurotoxicity. Studies concerning motor neuron disease, and any remaining duplicates were excluded. Physical modalities such as ultrasound, laser, and transcutaneous electrical nerve stimulation (TENS) were excluded from the search for the existing review about their role on nerve repair [24,25,26]. Zotero software was used to manage the citations and auto-eliminate duplicates.

### 2.3. Selection Criteria and Data Extraction

To identify eligible studies, two authors independently conceptualized keywords, and came to a consensus on which keywords to use for the search; then, each author independently assessed the articles. For quality assurance, study research and data extraction were repeated during the last update in September 2022. In the event of conflicting opinions, a consensus was reached after discussion between the authors. The selected full texts were then reviewed and included in the systematic review according to the Preferred Reporting Items for Systemic Reviews and Meta-analyses (PRISMA) statement [27], and following the Population, Intervention, Comparison, Outcome, and Study Design (PICOS) criteria [28] (Table 1 and Table 2).

### 2.4. Quality of the Results and Risk of Bias

Two independent authors assessed the included studies for the quality of study outcome (Table 3), and the risk of bias with SYRCLE’s tool [29]. The risk of bias elements included sequence generation, baseline characteristics, allocation concealment, blinding of investigators, random outcome assessment, blinding of assessor, incomplete outcome data, selective outcome reporting, and other sources of bias (Table 4). Their quality was low, unclear, or high for risk for each study analyzed by the authors.

**Table 1:** Physical exercise and instrumental therapy in peripheral neuropathy in animals: characteristics and outcomes of studies included in the systematic review.

Authors, Publication Year	Study Design, Sample (Species; Body Weight)	Groups	Peripheral Nerve Lesion	Intervention	Assessments, Parameters, Scales, Scores, Indices	Results
Ahlborn 2007 [30]	Randomized blinded study 16 female rats (C57BL/6J r) (weight unspecified)	- Electrically stimulated group: n.8 rats - Controlled sham-stimulated group: n.8 rats	Femoral nerve	Low-frequency electrical stimulation at 20-Hz once for one hour after 1 week from injury	Video-based motion analysis that allowed the precise evaluation of muscle function during locomotion	Brief electrical stimulation of femoral nerve led to accelerated locomotor recovery with respect to control group at 12 weeks (*p* = 0.063)
Asensio-Pinilla 2009 [11]	Comparative preclinical experimental study 45 female rats (Sprague–Dawley) (250 ± 300 g)	- Electrical stimulation group: 1 h, only immediately after injury: n.17 rats - Electrical stimulation group 2: 1 h daily for 4 weeks: n.8 rats - Electrical stimulation group 3: 1 h daily + treadmill running: n.10 rats - Group 4 treadmill running. n.10 rats	Sciatic nerve	Electrical stimulation (3 V, 0.1 ms at 20 Hz) and/or treadmill running (for 4 weeks, 5 m/min, 2 h daily)	Nerve conduction study: H reflex and algesimetry tests performed at 1, 3, 5, 7 and 9 weeks after surgery. Thermal nociception evaluated by a heat-radiation method using the plantar test	Combining electrical stimulation with treadmill significantly improved muscle reinnervation during the initial phase compared with running group (*p* < 0.05)
Boeltz 2013 [31]	Comparative preclinical experimental study 12 female rats (Sprague–Dawley and Lewis) (±250 g)	- Trained group: n.6 rats - Untrained group: n.6 rats	Tibial nerve	Moderate daily treadmill 5 days/week for 2 weeks, at a slow speed (10 m/min), for 1 h/day, beginning 3 days after transection and surgical repair	Nerve conduction study: H reflexes, kinematic parameters (limb angle, length during locomotion), M-response latency	Moderate daily exercise applied immediately after nerve injury is sufficient to promote axon regeneration, and restore muscle reflexes (*p* < 0.009 vs. untrained
Brown 1979 [32]	Preclinical comparative experimental study (weight unspecified)	- Stimulated nerves 42 ± 11 - Control non-stimulated muscles 50 ± 9	Peroneal nerve	Direct stimulation 100 Hz for 0.5 s/30 s or 150 Hz for 0.5 s/10s	Histological examination of nerve	Direct stimulation of a partially denervated muscle inhibits sprouting vs untreated muscles (*p* < 0.00003).
Brushart 2005 [33]	Randomized study 14 female rats (Sprangue-Dawley) (±250 g)	- Group 1: bilateral femoral muscle branches labelled with Fluoro Gold: n.3 rats - Group 2: injured nerves without electrical stimulation: n.6 rats - Group 3: injured nerves subjected to intraoperative electrical stimulation: n.5 rats	Femoral cutaneous branch	A total of 1 h of 20 Hz electrical stimulation	Histological examination of nerve	Electrical stimulation is thus highly effective at altering the pathway choices made by regenerating sensory axons, both decreasing projections to muscle nerve (*p* = 0.0282) and increasing those to cutaneous nerve (*p* = 0.0008)
Cobianchi 2010 [34]	Randomized blinded study 60 male rats (CD1) (40–45 g)	- Untrained group: n.23 rats - Runner group 1: from day 3 to day 7 post- injury: n.23 rats - Runner group 2: from day 3 to day 56 post-injury: n.14 rats	Sciatic nerve	- Short-lasting running (1 h/day for 5 days) - Long-lasting running (1 h/day for more than 5 days)	Sciatic nerve immunohistochemistry; Electronic Von Frey and Plantar test devices to measure mechanical and thermal, nociceptive withdrawal thresholds	Short-lasting treadmill running, by reducing the neuropathic pain symptoms and facilitating the regenerative processes of the injured nerve, has beneficial rehabilitative effects on the functional recovery after peripheral nerve injury reflexes compared to long-lasting groups (*p* < 0.05)
Cohan 1986 [35]	Comparative preclinical experimental study snails. A total of 143 growth cones (extension of a developing or regenerating neurite) from 21 neurons	- Stimulated snail neurons isolated in cell culture - Not stimulated snail neurons	Peripheral nerves	Electrical activity 45 mV, 10 msec	Rates of growth cone	Growth rates decreased from 12.5 ± 1.1 µm/hour before stimulation to 4.7 ± 1.4 µm/hour after stimulation (*p* < 0.002; 18 growth cones)
De Moraes 2018 [36]	Randomized study 30 male rats (BALB/c) (200– 300 g)	- Sham sedentary group: n.6 rats - Control group: sedentary for 14 days after nerve injury: n.6 rats - Control group: 42 days after nerve injury: n.6 rats - Control group: 70 d after nerve injury: n.6 rats - Exercises group: 7 d after nerve injury and 35 weeks of physical exercise: n.6 rats	Sciatic nerve	Swimming daily sessions in a glass tank with a 30 cm depth, water temperature at 32 ± 0.5 °C	Histological examination of nerve	Moderate swimming was a therapeutic resource for nerve regeneration. Nerve area and minimum diameter were significantly lower (*p* < 0.05) compared to control group
Einsiedel 1994 [37]	Comparative preclinical experimental study 32 rats (Sprague–Dawley) (400 g)	- Partially denervated and exercised animals: n.3 rats - Control partially denervated animals: n.4 rats - Unoperated control animals: n.25 rats	Sciatic nerve	Treadmill walking for 1.5 h/day and after 14 days walking a least 1 km/day	Fatigue index, vulnerability index, mean peak tetanic force, innervation ratio, time to peak of isometric twitch; twitch force; maximal tetanic force; axonal conduction velocity, weight muscle, CNAP, CSA	Increased motoneuron activity induced by treadmill walking is an important factor in determining the rate of motoneuron sprouting compared to unoperated animals (*p* <0.05)
Eisen 1973 [38]	Comparative preclinical experimental study n.22 rats (thy-1-YFP-H) (275 g)	- Immobilization group: n.7 rats - Contralateral immobilization group: n.7 rats - Control group: n.8 rats	Sciatic nerve	Contralateral immobilization 0–6 weeks	Histological examination of nerve: mean number of fibers/nerves, axon and fiber diameter	The differences in fiber diameter of nerves from limbs of non-immobilized animals compared to immobilized limbs and contralateral limbs are significant (*p* < 0.001)
English 2007 [39]	Comparative preclinical experimental study n.6 rats (NT-4/5 knockout) (weight unspecified)	- Electrical stimulation group: n.3 rats - Untreated group: n.3 rats	Sciatic nerve	One-hour application of electrical stimulation at 20 Hz at the time of surgical repair	Immunohisto-fluorescence	Electrical stimulation enhances axons regeneration in cut peripheral nerves, independent of neurotrophin, but dependent on stimulation of trkB. Among electrical stimulation neurons, a significant increase in the proportion of neurons as immunoreactive to BDNF was recorded compared to untreated group (*p* < 0.01)
Florence 2001 [40]	Comparative preclinical experimental study n.6 monkeys (Macaca radiata) (weight unspecified)	- Sensory manipulation group: n. 5 - Untreated group: n.6 - Restricted sensory experience: n.1	Median nerve	Rehabilitation involving sensory retraining	Electrophysiological mapping studies	In the monkeys reared without sensory enrichment during recovery, there were significantly larger and multiple fields compared to the sensory enriched monkeys (*p* < 0.05)
Gardiner 1984 [41]	Comparative preclinical experimental study n.49 rats (female Sprague–Dawley) (weight unspecified)	- Training Group: n.23 rats5 - Control group non-trained: n.26 rats	Sciatic nerve	Treadmill immediately after nerve injury for 10 weeks for 1 h, at a speed of 26.8 m/min and an inclination of 15%	Body and muscle weights, twitch and tetanic contraction	Physical exercise enhances short-term sprouting of fast muscle motoneurons compared with untreated group (*p* < 0.05)
Gardiner 1986 [42]	Randomized study n.50 female rats (Sprague–Dawley) (180–200 g)	- Partial denervation untreated group: n.12 rats - Partial denervation treated group: n.15 rats - Healthy controls: n.13 rats - Treated control without denervation: n.6 rats - Trained group for 28 days: n.4 rats	Sciatic nerve	Daily program of increased activity, including grid climbing and voluntary wheel exercise, for 14 days, denervation at 5th day.	CSA, maximum tetanic tension	Muscle tetanic tension of partially denervated muscles in partial denervation of one hindlimb group and partial denervation of one hindlimb plus daily exercise were significantly (*p* < 0.05) larger than L5-evoked tension in left control muscles, and significantly (*p* < 0.05) lower than L4 plus L5-evoked control tension
Geremia 2006 [16]	Comparative preclinical experimental study n.76 female rats (Sprague–Dawley) (220–240 g)	- Group 1: sham stimulation: n.13 rats - Group 1: after 1 h of stimulation: n.19 rats - Non-stimulated group: n.7 rats - Total of 1 h of electrical stimulation: n.19 rats - Total of 3 h of electrical stimulation: n.6 rats - Total of 1 day of electrical stimulation: n.3 rats - Total of 7 days of electrical stimulation: n.4 rats - Total of 14 days of electrical stimulation: n.5 rats	Femoral nerves	20 Hz continuous electrical stimulation immediately after surgical repair	Hybridization quantification and analysis; immunohistochemistry	Electrical stimulation of 1 h led to a significant increase in DRG neurons regenerating into cutaneous and muscle branches, significantly increased the numbers of neurons that regenerated axons compared with the other group (*p* < 0.05), increased expression of GAP-43 in the regenerating neurons and of BDNF compared with the other group (*p* < 0.001)
Gomez-Pinilla 2002 [13]	Randomized study 10 male rats (Sprague–Dawley) (weight unspecified)	- Exercise group: n.5 rats - Sedentary group: n.5 rats	Peripheral neuropathy	Wheel running for 7 days	Levels of neurotrophins and neurotropic factors: BDNF and its signal transduction receptor (trkB)	Voluntary exercise increased the expression of several molecules associated with the action of BDNF on synaptic function and neurite outgrowth in the lumbar region of the spinal cord and soleus muscle comparing sedentary group (*p* < 0.01)
Herbison 1973 [43]	Comparative preclinical experimental study 15 female rats (Wistar) (200 g)	- Controlled Group I not denerved: n.5 rats - Group II undergone tenotomy of two synergists 3 weeks after denervation: n.5 rats - Group III sham-operated sedentary control, without tenotomy: n.5 rats	Sciatic nerve	Overwork (induced in soleus and plantaris by tenotomy of synergistic muscles) after 72 h, 1, 2, 3, 4, 6 weeks from denervation	Muscle weights, protein content, and conduction latencies, fiber diameters	Overwork within the period of reinnervation may be more beneficial than when initiated before this event. In the Group of tenotomy 3 weeks after denervation, the muscle weights, absolute amount of sarcoplasmic, myofibrillar, and stromal proteins and Type I and II fiber diameters of the soleus and plantaris were greater (*p* < 0.05) than control values
Herbison 1974 [44]	Comparative preclinical experimental study 40 rats (Wistar) (200–215 g)	- Sedentary control group A: n.8 rats - Early reinnervation group groups B started 3 weeks after denervation swimming 1 h daily for 5 days a week for 3 weeks: n.8 rats - Group C started 3 weeks after denervation exercised 2 h daily for 3 weeks: n.8 rats - Group D started exercise 4 weeks after denervation and trained for 2 weeks swimming 1 h each day: n.8 rats - Late reinnervating group E started exercise 4 weeks after denervation and trained for 2 weeks. Swimming 2 h each day: n.8 rats	Sciatic nerve	Swimming 3–4 weeks after denervation for one or two hours each day for 3–4 weeks	Histological examination of nerve: fiber diameter and composition of fiber types in reinnervating soleus and plantaris muscles, total proteins	Intense swimming (2 h every day) does not enhance the repair of reinnervation muscle, and a high workload may be hazardous in the early phase of reinnervation. Moreover, Group C total protein concentration was significantly lower (*p* < 005) than that of the remaining four groups
Herbison 1980 [45]	Comparative preclinical experimental study 21 groups of female rats (Winstar) (200–225 g)	- Healthy controls: n.7 groups - Sciatic nerve crush-denervated controls: n.5 groups - Crush-denervated casted: n.5 groups - Crush-denervated exercised: n.4 groups	Sciatic nerve	Treadmill 27m/min at 35% grade and bilateral cast immobilization I hind limbs were started 2–3 weeks after sciatic nerve crush 5 with a current of 15–20 V	Isometric twitch/tension, time to peak tension.	The extremes of activity or inactivity retarded, but did not prevent, the recovery of the slow more than the fast muscle during reinnervation. Moreover, the tetanic tensions of the crush-denerveted muscles were significantly different from the crush-denervated control values at 6 weeks after crush; the soleus was 20.6% less (*p* < 0.05) and the plantaris was 23.5% greater (*p* < 0.01) than the crush-denervated control values
Herbison 1986 [46]	Comparative preclinical experimental study n.30 female rats (Wistar) (200–225 g)	- Healthy control group: n.6 rats - Partial denervated group: n.6 rats - Electrical stimulated group for 2 h: n.6 rats - Electrically stimulated group for 4 h: n.6 rats - Electrically stimulated group for 8 h: n.6 rats	Sciatic nerve	Electrical stimulation 4 ms, 2–4 mA current distributed at 10 pulses per second stimulated for 2, 4, 8 h per day, 5 days per week for 6 weeks	Electric study, muscle weight, twitch and tetanic tension, fiber area, contraction time	Chronic stimulation of intact axons of partially denervated muscle increases the muscle weight and tension of the electrically stimulated muscle. Moreover, the weights of the muscles of the bilateral partial nerve section groups compared with their respective normal control muscles showed significant atrophy (*p* < 0.01)
Hines 1942 [47]	Comparative preclinical experimental study Albino rats (weight unspecified)	- Group A paralyzed limb for 5 h daily in water at 112 to 117 F - Group B - paralyzed limb for 5 h daily in ice water	Tibial nerve	Forced activity	Muscle creatine content, isometric tension, muscle weight	Activity improves the rate and extent of the recovery from peripheral nerve paralysis. After 21 days, there was no difference in the strength and weight of the muscle from the animals in the immobilized groups (*p* > 0.05)
Huang 2012 [17]	Randomized blinded study n.140 young male rats (Sprague–Dawley) (weight unspecified)	- Conductive scaffold + electrical stimulation group: n.70 rats - Non-conductive scaffold + electrical stimulation group: n.70 rats	Sciatic nerve	Intermittent electrical stimulation (3 V, 20 Hz) every two days for 8 times, assessed at 4, 8, 12 weeks Direct current or alternating current	Expression of regeneration-associated genes, SFI, modified Sticky Tape Test, morphometric analysis, atrophy, protein levels	Electrical stimulation accelerates nerve regeneration and promotes functional recovery. Moreover, the protein levels of S-100, BDNF, P0 and Par-3 were significantly upregulated in the conductive scaffold + electrical stimulation group compared to non-conductive scaffold + electrical stimulation group (*p* < 0.05)
Ilha 2008 [48]	Randomized study n.37 male rats (Wistar) (280–330 g)	- Control group: n.8 rats without sciatic crush and unexercised - Sedentary group: n.7 rats - Endurance trained group: n.7 rats - Resistance trained: n.7 rats - Endurance-resistance trained: n. 8 rats	Sciatic nerve	Endurance on a treadmill (from 20 to 60′ in 4 weeks) + warmup (running for 5′) 5 sessions per week, once a day during 5 weeks + Resistance Exercise (Climbing a 1 m-long ladder) A total of 3 sessions per week with 48 to 72 h of rest between sessions for 5 weeks	Histological and Morphometric Nerve Studies, SFI	Endurance exercise improves sciatic nerve regeneration and resistance exercise. Endurance-resistance training may delay functional recovery and does not alter sciatic nerve fiber regeneration. Moreover, the SFI values of experimental groups were significantly lower than those of the control group (*p* < 0.001)
Irintchev 1990 [49]	Comparative preclinical experimental study n.15 rats (C57Bl/6J) (300 g)	- Trained group during both the denervation period and during reinnervation: n.9 rats - Trained group after the last nerve freezing: n.7 rats - Trained group during the denervation period only: n.5 rats, 40 days after reinnervation	Sciatic nerve	Running 7–8 weeks in wheels (5.8 km + 1.5 S.D.) during the time of denervation and reinnervation period	Tetanic muscle force, muscle weight	Tetanic muscle force reached on average 72% of contralateral muscles after 5–10 months, (*p* < 0.01) and 87% of unoperated animals after 10 months (*p* < 0.05)
Irintchev 1991 [50]	Comparative preclinical experimental study n.10 female rats (NMRI) (weight unspecified)	- Runner group: n.5 rats - Sedentary group: n.5 rats	Sciatic nerve	Running wheels 39 weeks, 14–18 weeks after the nerve injury	Tetanic muscle force, isometric contraction measurements, muscle weight	Physical exercise during progressive muscle atrophy is effective and has significant and enduring impact on muscle recovery after reinnervation There were highly significant effects of exercise on nerve damage (*p* = 6–7% for the F-ratio)
Jaweed 1982 [51]	Comparative preclinical experimental study n.60 female rats (Wistar) (200–225 g)	- Stimulated group: n.30 rats - Healthy controlled Group: n.30 rats	Sciatic nerve	Low-frequency electrical stimulation (2- to 4-mA pulses at 4 ms duration) at 10 Hz continuously 8 h daily for 10, 15, 20, 25, 30 days	Isometric twitch contraction, muscle weight	The effectiveness of long-term (200 to 240 h) direct, low frequency (10 Hz) electrical stimulation. In normal muscle, 25 and 30 days of electrical stimulation produced significant results (*p* < 0.05)
Kao 2013 [20]	Randomized study n.50 diabetic male rats (Sprague–Dawley) (250–300 g)	- Healthy control group: n.10 rats - Electrical stimulation group at 0 Hz: n.10 rats - Electrical stimulation group at 2 Hz: n.10 rats - Electrical stimulation group at 20 Hz: n.10 rats - Electrical stimulation group at 200: n.10 rats	Sciatic nerve	Percutaneous electrical stimulation 1 mA at 0, 2, 20, or 200 Hz, started after 1 week from the injury for 3 weeks	Morphometric analysis of axonal regeneration and remyelination; electrophysiological recordings of CMAPs for the conductive velocity, peak amplitude, area, latency 4 weeks postoperatively: macrophage density, CGRP area ratio	High-frequency electric stimulation could be necessary to heal the diabetic peripheral nerve. Larger nerve conductive velocity, amplitudes, and areas of the MAPs and shorter latencies were seen as the frequency of electrical stimulation was increased, where the differences of all of these parameters between the electrical stimulation groups at 0 Hz and 200 Hz reached significance at *p* < 0.05
Kim 1998 [52]	Randomized study n.33 rabbits (New Zealand white) (2000–2500 g)	- Continuous passive motion group: n.10 - Immobilization controlled group: n. 13 - Sham-operated: n.10	Medial popliteal nerve	Continuous passive motion for 14 days	Nerve conduction study at 100 days (nerve conduction velocity, fiber density, and diameter, weight of soleus)	Continuous passive motion after nerve repair induces regeneration. The mean nerve conduction velocity was significantly lower in the two treatment groups than in the control groups (*p* = 0.0001)
Liao 2017 [19]	Randomized study (species and weight unspecified)	- Sedentary control group: n.10 rats - Swam group (10 min/3 times/week): n.10 rats - Swam group (20 min/3 times/week): n.10 rats - Swam group (30 min/3 times/week) (n=10 each): n.10 rats	Sciatic nerve	Swimming	Axon regeneration, electrophysiological parameters, muscular weights, macrophage infiltration, CGRP	Moderate swimming significantly improved CGRP-related axonal regeneration. Total nerve regeneration area of the swam group (10 min/3 times/week) was significantly elevated to approximately two-fold more than that of the sedentary control group (*p* < 0.05)
López-Álvarez 2015 [53]	Randomized blinded study rats (Sprague–Dawley) (240–300 g)	- Group 1: Daily sessions for 5 consecutive days, from 3 to 7 days postinjury: n.8 rats - Group 2: Continued physical exercise from 10 to 14 days postinjury: n.7 rats - Group 3: Untrained control group: n.8 rats	Sciatic nerve	A total of 1 h running, starting at a locomotion speed of 10 cm/s and increasing 2 cm/s every 5 min, until a maximal speed of 32 cm/s	Thermal and mechanical thresholds Sensory PGP-IR fibers, BDNF, GAP43, NGF expression, CGRP neurons in the L3 DRG and KCC2 dephosphorylation in the dorsal horn, microglial activation	Recodification of spontaneous neural activity after peripheral nerve injury by specific graded intensity exercises may be a potent neurorehabilitation tool to prevent neuropathic pain. The expression of BDNF in microglia was greatly increased in untrained injured rats after sciatic nerve lesion compared to that of the untrained group (*p* < 0.0001)
Love 2002 [54]	Comparative preclinical experimental study n. 9 rats (species and weight unspecified)	- Electrical stimulation group: n.6 rats - Sham group: n.3 rats	Tibial nerve	Electrical stimulation of 12 mA for 7 days (20 or 100Hz)	Fluorescent Measurements of Tension and Motor Unit Size; Fluorescent Labeling	Muscle stimulation reduces sprouting by removing the means by which sprouts navigate to denervated end plates, i.e., terminal Schwann cells bridges. The number of end plates reinnervated by nodal sprouts was 19 ± 8% in sham-stimulated muscles and 14 ± 3% in stimulated muscles (*p* < 0.18)
Marqueste 2003 [6]	Randomized study n.36 rats (Sprague–Dawley) (weight unspecified)	- Group C: healthy untreated control animals: n.10 rats - Group LS: Animals with nerve lesion and suture: n.14 rats - Group LSE: Animals with lesion, suture, and chronic muscle rehabilitation by electrostimulation: n.12 rats	Peroneal nerve	Biphasic electro-myo-stimulation and exercise 5 days/week for 10 weeks	Monopolar tungsten electrode, CNAP	Chronic muscle electrostimulation partially favors the recovery of muscles, rehabilitation by treadmill running also efficiently induced a better functional muscle afferent recovery. When twitches were induced by muscle stimulation, CT significantly (*p* < 0.01 and *p* < 0.001) decreased in the LS and LSE groups
Marqueste 2006 [55]	Randomized study n.56 female rats (Sprague–Dawley) (300–350 g)	- Unoperated control group: n.10 rats - Untreated nerve repair: n.14 rats - Monophasic rectangular current (LSEm): n.10 r ats - Biphasic modulated current (LSEb): n.12 rats	Peroneal nerve	Monophasic or biphasic electro-myo-stimulation from 4 Hz to 75 Hz for 10 weeks	Muscle weight, Twitch characteristics, fatigue index, protein	Muscle electrostimulation following denervation and reinnervation tends to restore size (muscle atrophy was reduced in LSEm and absent in LSEb groups) and functional and histochemical properties during reinnervation better than unstimulated muscle. *p* < 0.001 indicated that the fatigue index significantly differed from that of controlled rats
Martins 2011 [56]	Randomized blinded study n.56 rats (Wistar) (250–280 g)	- Sham group: n.8 rats - Sham-operated+ Anesthesia group: n.8 rats - Sham-operated + ankle joint mobilization group: n.8 rats - Crush-operated group: n.8 r - Crush-operated + anesthesia group p: n.8 rats - Crush-operated + ankle joint mobilization group: n.8 rats - Crush + ankle joint mobilization group: n.8 rats	Sciatic nerve	A total of 15 sessions every day of joint mobilization	Morphological analysis and immunoreactivity of CD11b/c and GFAP, SFI	Mobilization produces an anti-hyperalgesia effect and peripheral nerve regeneration. Mechanical and thermal hyperalgesia and motor performance deficit were detected in the Crush + Anesthesia group (*p* < 0.001), which was significantly decreased after joint mobilization (*p* < 0.001). In the morphological analysis, the Crush + Anesthesia group presented reduced myelin sheath thickness (*p* < 0.05), but the joint mobilization group presented enhanced myelin sheath thickness (*p* < 0.05) Peripheral nerve injury increased the immunoreactivity for CD11b/c and GFAP in the spinal cord (*p* < 0.05), and joint mobilization markedly reduced CD11b/c and GFAP immunoreactivity (*p* < 0.01)
Martins 2017 [57]	Blinded study n.40 male rats (swiss) (250–300 g)	- Sham-operated: n. 8 rats - Crush control non-exercised: n.8 rats - Nerve crush + eccentric exercise 6 m/min: n.8 rats - Nerve crush + eccentric exercise 10 m/min: n.8 rats - Nerve crush + eccentric exercise 14 m/min: n.8 rats	Sciatic nerve	Treadmill for 30 min at a speed of 6, 10, or 14 m/min with–16° slope, 5 days per week, over 8 weeks. Exercises began on the second post-operative week	Grip strength test, SFI	Exercised groups presented less neuropathic pain-like behavior and better functional recovery than non-exercised groups. Biochemically, exercise reduced TNF-α in the muscle and increased sciatic nerve IGF-1 levels in sciatic nerve crush. SFI value of the regular eccentric exercise groups were significantly better than those of the crush non-exercise group (*p* < 0.05)
Michel 1989 [58]	Comparative preclinical experimental study 70 female rats (Sprague–Dawley) (200–220 g)	- Healthy untreated control group: n.15 rats - Overload group: n.15 rats - Partial denervation group: n.20 rats - Overload plus partial denervation group: n.20 rats	Sciatic nerve	Overload for 37 days	Body and plantaris weight, cross-sectional area, half-relaxation time, and maximum tetanic tension, fatigue index	Neuromuscular adaptation in response to compensatory overload does not favor the functional recovery from a partial denervation lesion. Significant main overload and partial denervation effects and interactions (*p* < 0.05) of groups compared with the control group
Molteni 2004 [7]	Blinded study n.12 rats (species and weight unspecified)	- Exercise group: n.6 rats - Sedentary animal group: n.6 rats	Sciatic nerve	A total of 3 or 7 days of exercise	Analysis of regeneration; immunofluorescence; isolation of RNA and Real-Time Quantitative RT-PCR	Voluntary enhanced regrowth of axons after nerve injury. Differences in length of 3 and 7 days vs. 0 and 7 days vs. 3 days were statistically significant (*p* < 0.0001 and *p* < 0.01, respectively)
Pachter 1989 [8]	Comparative preclinical experimental study n.18 rats (Wistar) (weight unspecified)	- No-denervated untreated control group: n.6 rats - Exercised denervated group: n.6 rats - Non-exercised denervated group: n. 6 rats	Peroneal nerve	A total of 4 days (2 h/day) of physical exercise	Isometric contractile properties, endplate ultrastructure	Denervated muscles exercised 4 days before reinnervation can preserve the structure of the endplate, enhance reinnervation and sprouting at the endplates after 11 days of denervation. The postsynaptic area and endplate were decreased compared with the control group (*p* < 0.05)
Sabatier 2008 [12]	Comparative preclinical experimental study n.19 rats (Thy-1-YFP-H) (weight unspecified)	- Group 1: low-intensity continuous physical exercise (60 min): n.5 rats - Group 2 low-intensity interval physical exercise, faster speed (20 m/min) for 2 minutes, separated by 5-min rest, 2 repetitions: n.4 rats - Group 3 high-intensity, faster speed (20 m/min) for 2 minutes, separated by 5-min rest, 4 repetitions: n.3 rats - Group 4 high-intensity faster speed (20 m/min) for 2 minutes, separated by 5-min rest, 10 repetitions: n.4 rats - Group 5 faster speed (10 m/min) for 2 minutes, separated by 5-min rest, 10 repetitions: n.3 rats	Sciatic nerve	Two weeks of treadmill, 5 days per week for 2 weeks	Tissue Harvesting and Microscopy	Treadmill exercise enhances axon regeneration in the peripheral nervous system. The sprouting index was significantly increased in all high-intensity groups (*p* ≤ 0.05)
Sarikcioglu 2001 [59]	Comparative preclinical experimental study n.36 rabbits (weight unspecified)	- Healthy control group: n.6 - Nerve clamped exercises group: n.30	Sciatic nerves	Swam 10 min/day for 10 days in a pool at 37 °C tap water	HRP neuro-histochemistry and modified Pal-Weigert methods	Exercise is effective for axonal regeneration in the 4^th^ regeneration week. There was no myelinated fiber in the sedentary group, and there was a significant difference between exercise trained and sedentary groups (*p* < 0.05)
Seburn 1996 [60]	Randomized study n.73 male rats (Sprague–Dawley) (250–300 g)	- Running group: n.36 rats - Sedentary group: n.37 rats	Popliteal nerve	Running	Motor unit tetanic force	Daily locomotor activity can enhance the tension-generating capacity of chronically enlarged motor units compared to sedentary group (*p* < 0.05)
Seo 2006 [61]	Randomized blinded study n.160 male Sprague–Dawley rats (220–240 g)	- Treadmill group - Sedentary group	Sciatic nerve	Treadmill walking for 10 min/day for 2 days prior to sciatic nerve injury	SFI, Western blotting and immunofluorescence staining	Treadmill promoted axonal regeneration. Differences in SFI values among the groups were statistically significant (*p* < 0.003)
Seo 2009 [21]	Randomized blinded study 108 male rats (Sprague–Dawley) (200–220 g)	- Intact control group: n.6 rats - Low-intensity Treadmill: n.6 rats - High-intensity treadmill: n. 6 rats - Sedentary group with sciatic nerve injury: n.30 rats - Treadmill group with sciatic nerve injury: n.60 rats	Sciatic nerve	- Low-intensity treadmill 8 m/min for 30 min twice a day for 14 days - High-intensity treadmill 36 m/min for 30 min twice a day for 14 days	Levels of neurotrophins and neurotropic factors: expression levels of GAP-43 mRNA	Increased ERK1/2 activity in Schwann cells may play an important role in treadmill-mediated enhancement of axonal regeneration in the injured peripheral nerve. Protein levels in the treadmill groups were significantly higher than in sedentary controls (*p* < 0.01)
Sinis 2008 [62]	Randomized blinded study n.48 female rats (Fast Blue) (175–200 g)	- Healthy untreated control group 1: n.12 rats - Group 2: transection and suture of median nerve only: n.12 rats - Group 3: transection-suture and manual stimulation: n.12 rats - Group 4: transection and suture and handling: n.12 rats	Facial nerve, median nerve	Manual stimulation	Restoration of grasping force, degree of collateral axonal branching, pattern of reinnervation of the motor endplates, index of axonal branching	Manual stimulation is beneficial in motor nerve injury, not in mixed nerves. It did not influence the degree of axonal sprouting or the extent of poly-innervation of motor endplates (*p* < 0.05)
Skouras 2009 [63]	Comparative preclinical experimental study n.64 female rats (Wistar) (175–200 g)	- Untreated control group: n.16 rats - Sham stimulated group: n.16 rats - Electrical stimulation group: immediately after facial nerve transection but prior to end-to-end suture: n.16 rats - Manual stimulation group: n.16 rats	Facial nerve	- Electrical stimulation (20 Hz) for 1 h prior to nerve reconstruction - Manual stimulation, daily rhythmic stroking of the whisker pads for 4 months	Video-based motion analysis, analysis of vibrissae motor performance	Electrical stimulation did not improve functional outcome and failed to reduce the proportion of poly-innervated motor end-plates. By contrast, manual stimulation restored normal whisking function and reduced poly-innervation (*p* < 0.05).
Sobral 2008 [64]	Comparative preclinical experimental study n.20 male rats (Wistar) (229.05 ± 18.02 g)	- Control group: n.5 rats - Denervated group: n.5 rats - Denervated+ exercise+ cage DEC: n.5 rats - Denervated+ cage+ exercise DCE: n.5 rats	Sciatic nerve	Running at a speed=8m/min, inclination = 0%, 30 min/day, for 14 days. DEC group started exercise 24 h after the nerve injury. DCE group started on the 14^th^ day after the injury, assessment at 7^th^, 14^th^, 21^st^ and 28^th^ days after the operation	Axon and fiber diameter, myelin thickness, SFI	The treadmill exercise, during the immediate and late phase of nerve regeneration after crushing the sciatic nerve of rats, did not influence axonal budding, degree of maturation of the regenerated fibers or the functionality of the reinnervated muscles. The number of regenerated axons in denervated + cage + exercise groups was greater than in the others (*p* < 0.05)
Soucy 2013 [65]	Randomized blinded study n.8 female rats (Sprague–Dawley) (135–155 g)	- Sedentary Group: n.8 rats - Exercise group: n.8 rats	Sciatic nerve	Voluntary Motor Activity 9 days of daily handling and mild treadmill exercise. A total of 60 min at a speed of 30 m/min, at a 5% incline	Histological examination of nerve: regeneration rate of axons	Increased activity has no effect on axon regeneration rate, but may be detrimental to the reinnervation process. Significant effect of exercise, crush, and interaction (*p* < 0.05) were detected at force integral index measured at force integral index measured at frequencies of 100–400 Hz
Tam 2001 [66]	Randomized blinded study n.55 female rats (Sprague–Dawley) (180–200 g)	- Group 1 normal caged activity: n.20 rats - Group 2 running exercise on wheels: n.11 rats - Group 3 electrical stimulation: n.12 rats - Control group: n.12 rats	Sciatic nerves	- Running exercise on wheels, 8 h daily, for 4 weeks immediately after denervation started - Electrical stimulation 20 Hz for 8 h daily, for 4 weeks, immediately after denervation started	Electrophysiological and histochemical examination, CSA	Increased neuromuscular activity is not recommended as rehabilitation immediately after motoneuron injury or in the early stages of motoneuron disease. MU twitch forces were significantly larger than those of the control (*p* < 0.001). The shift in the MU twitch force distributions was much less but significant (*p* < 0.01) for moderately denervated muscles
Teodori 2011 [67]	Randomized study n.20 male rats (Wistar) (220 ± 12 g)	- Group 1 (CS1) swim immediately after crush nerve injury: n.5 rats - Group 2 (CS14) begins to swim 14 days after injury: n.5 rats - Group 3 (C) injured, not submitted to swimming: n.5 rats - Group uninjured, submitted to swimming: n.5 rats	Sciatic nerve	Swimming	SFI, axon number and diameter, fiber diameter and numbers, myelin thickness	After 30 days, the number of axons in CS1 and CS14 was lower than in C (*p* < 0.01). The diameter of axons and nerve fibers was larger in CS1 (*p* < 0.01) and CS14 (*p* < 0.05) than in C, and myelin sheath thickness was lower in all crushed groups (*p* < 0.05). There was no functional difference between CS1 and CS14 (*p* > 0.05)
Udina 2010 [68]	Comparative preclinical experimental study n.30 female rats (Sprague–Dawley) (weight unspecified)	- Passive exercise group: n.10 rats - Active exercise group: n.10 rats - Untreated group: n. 10 rats	Sciatic nerve	- A total of 30 min of passive exercise at a cycling rate of 45 rpm separated by 10 min of rest, for 1 month, at day 5 post-operation - Active (treadmill) exercise for 1 h/day 30 min at a speed of 4.6 m/min, with a 10-min rest period	Nerve conduction study: Latency, M amplitude, H/M ratio, MEP/M ratio	Exercise increases trophic factor release to act on regenerating axons and to modulate central neuronal plasticity. MEP/M amplitude ratio during follow-up in rats untreated and treated with passive and active exercise for gastrocnemius, tibialis anterior, and plantar muscles is significantly different than the untreated group (*p* < 0.05).
Van Meeteren-Wiegant 1997 [69]	Randomized blinded study n.20 rats (Wistar) (200–220 g)	- Group 1: n.10 high-active rats - Group 2: n.10 low-active rats	Sciatic nerve	Locomotor activity in the open field	SFI, withdrawal reflex	Existence of a relationship between individual behavioral characteristics and sensory recovery of nerve function following crush lesion in rats. Recovery of motor function revealed no significant differences between both groups, whereas recovery of sensory function in active rats was significantly more rapid than that of the low active rats (*p* = 0.01)
van Meeteren– Gispen 1997 [70]	Randomized blinded study n.20 male rats (Wistar) (140–160 g)	- Sedentary control: n.10 rats - Active control: n.10 rats	Sciatic nerve	Physical exercises	SFI, electrophysiologic findings: motor nerve conduction velocity	Beneficial effects of 24 days of exercise after crush persist in the late phase of peripheral nerve recovery. The motor nerve conduction velocity, as measured in the late phase of recovery, was significantly better in the trained group than in the control group (*p* < 0.01).
Werning 1991 [71]	Comparative preclinical experimental study n.30 male rats (CBA/J) (weight unspecified)	- Group 1: exercises once for a total period of 9 h: n.24 rats - Group 2: the same exercise repeated 8–10 times at intervals of 3–5 days: n.6 rats	-	Motor-driven treadmill, for total period of 9 h (3 x 3 h) with 30 min rest periods in between, 14 m min^−1^, slope of 6 degrees	Histological examination of nerve: chronic signs of damage: split fibers, central nuclei	The incidence of sprouting was significantly elevated 3–21 days after a single exercise (*p* < 0.01), and more so after repeated running (*p* < 0.01)

Years, y; Sensory Amplitude, SNAP; Motor Amplitude, CMAP; Physical Activity Scale for the Elderly, PASE; Nerve Action Potential Amplitude, CNAP; Repetitions, reps; Extracellular signal-regulated Kinase ½, ERK1/2; Sciatic Nerve Function Index, SFI; Cross-Sectional Area, CSA; Grams, g; Growth-associated Protein 43, GAP-43; Brain-Derived Neurotrophic Factor, BDNF; Calcitonin Gene-Related Peptide, CGRP; Glial fibrillary acidic protein, GFAP.

**Table 2:** Physical exercise in peripheral neuropathy in humans: characteristics and outcomes of studies included in the systematic review.

Authors, Year	Study Design	Sample Size, y	Neuropathy Characteristics	Rehabilitation and Instrumental Physical Therapy	Outcomes Measure	Results
Gordon 2009 [72]	Randomized control trial	A total of 21 subjects: 8 males, 13 females; 56 ± 17 y - Group 1 (FES): 11 subjects - Group 2 (control): 10 subjects	Post-surgical median nerve compression	A total of 1 h 20 Hz of bipolar FES	Nerve conduction studies: MUNE and NCS; Purdue Pegboard Test, Semmes Weinstein Monofilaments, Levine’s Self-Assessment Questionnaire	The stimulation group had significant axonal regeneration 6–8 months after surgery when the MUNE increased to 290 ± 140 motor units from 150 ± 62 MU at baseline (*p* < 0.05). In comparison, MUNE did not significantly improve in the control group (pN0.2). Terminal motor latency significantly accelerated in the stimulation group but not the control group (*p* > 0.1).
Inoue 2011 [73]	Controlled experimental study	A total of 7 subjects, elderly men - Neurapraxia 2, - Axonotmesis 4, - Neurotmesis 1 - A total of 5 peroneal nerves, - A total of 1 axillary nerve, - A total of 1 ulnar nerve	Peripheral peroneal: n.5, axillary: n.1, ulnar: n.1 neuropathies	Self-guided rehabilitation	EMG, AROM for ankle and great toe	Complete functional recovery was observed in neurapraxia and partially in axonotmesis, and others showed reinnervation. An EMG examination revealed fibrillation potential (denervation potential) and reinnervation potential
Kluding 2012 [74]	Randomized blinded control trial	A total of 17 subjects 8 males/9 females; 58.4 ± 5.98 y; (duration of diabetes 12.4 ± 12.2 y)	Diabetic peripheral neuropathy	In all, a 10-week exercise program with aerobic and strengthening exercises	Skin biopsy, QST of vibratory detection threshold, cooling detection threshold, and heat/pain threshold VAS, MNSI, IENF	Significant reduction in pain (*p* = 0.05), neuropathic symptoms (*p* = 0.01), and increased intraepidermal nerve fiber branching (*p* = 0.008) from a proximal skin biopsy were noted following intervention
Lange-Maia 2016 [75]	Controlled experimental study	A total of 328 subjects, old men, 78.8 ± 4.7 y	Peripheral neuropathy	A total of 7 days of walking, strenuous moderate and light activities, strengthening exercises, lawn work and gardening occupational activities	Automated neurodiagnostic instrument motor and sensory latency, nerve function, CMAP, SNAP, F-wave latency PASE	Improvement in peripheral neuropathy was modestly associated with daily vigorous physical exercise in older men. Better motor latency was associated with higher PASE scores (*p* < 0.01)
Mennen 2002 [76]	Controlled experimental study	A total of 56 subjects 52 males, 4 females -	Brachial plexus, ulnaris, medianous, radialis, digitalis, popliteus nerve lesion	Rehabilitation including sensory re-training and motor contraction exercises	EMG, MRC	End-to-side nerve suture and rehabilitation restores function, replace nerve grafting
Piccinini 2020 [77]	Randomized blinded control trial	A total of 38 subjects, 21 males, 17 females 37 ± 21 y	Interosseous, abductor digiti, extensor digitorum communis, brachioradialis, vastus lateralis and medialis, biceps femoris, gastrocnemius, tibialis anterior, peroneus longus	FES 150 ms, 1 Hz, 0.5 mA	MRC, strength with dynamometer, fibrillation potentials	FES improved in terms of clinical and neurophysiological parameters (MRC: *p* < 0.001 after FES)
Wong 2015 [78]	Randomized blinded Control Trial	A total of 36 subjects, 38.3 ± 39.3 - Group 1: 18 - Group 2: 18	Post-surgery after complete digital nerve transection	FES 1 h, 20 Hz	DASH, pressure threshold and quantitative small-fiber sensory testing	Post-surgical FES enhanced sensory reinnervation in patients who sustained complete digital nerve transection. Although there was a trend of greater functional improvements in the ES group, it was not statistically significant (*p* > 0.01)

Years, y; Sensory Amplitude, SNAP; Motor Amplitude, CMAP; Physical Activity Scale for the Elderly, PASE; Visual Analogue Scale, VAS; Michigan Neuropathy Screening Instrument, MNSI; Intraepidermal Nerve Fiber, IENF; Nerve Conduction Studies, NCS; Quantitative Sensory Testing, QST; Functional Electrical Stimulation, FES; Motor unit number estimation, MUNE; Disability of Arm, Shoulder and Hand questionnaire, DASH; Electromyography, EMG; Low-level laser therapy, LLLT.

**Table 3:** Quality of evidence of studies.

Quality Assessment					Summary of Findings	Quality of Evidence
N° of Studies	Limitations	Inconsistency	Indirectness	Publication Bias	Characteristics of	
60 studies	No significant limitations	No serious inconsistency	No serious indirectness	Unlikely	Population: humans or animals with neuropathic impairment Intervention: physical exercise or electrical stimulation Comparison: untreated group or treated with other therapy program Outcomes: changes in electrodiagnostic, muscle characteristics, specific indices and scales	Moderate–High

**Table 4:** Risk of bias summary for each included study.

Authors, Year	Random Sequence Generation	Allocation Concealment	Blinding Participants	Blinding of Outcome Assessment	Incomplete Data	Selective Reporting	Other Bias	Risk of Bias
Ahlborn 2007 [30]	+	+	+	+	+	+	+	Low risk
Asensio-Pinilla 2009 [11]	-	-	-	-	+	+	+	Moderate risk
Boeltz 2013 [31]	-	-	-	-	+	+	+	Moderate risk
Brown 1979 [32]	-	-	-	-	-	+	+	High risk
Brushart 2005 [33]	+	+	-	-	+	+	+	Low risk
Cobianchi 2010 [34]	+	+	+	+	+	+	+	Low risk
Cohan 1986 [35]	-	-	-	-	+	+	+	Moderate risk
De Moraes 2018 [36]	+	+	-	-	+	+	+	Low risk
Einsiedel 1994 [37]	-	-	-	-	+	+	+	Moderate risk
Eisen 1973 [38]	-	-	-	-	+	+	+	Moderate risk
English 2007 [39]	-	-	-	-	+	+	+	Moderate risk
Florence 2001 [40]	-	-	-	-	+	+	+	Moderate risk
Gardiner 1984 [41]	-	-	-	-	+	+	+	Moderate risk
Gardiner 1986 [42]	+	+	-	-	+	+	+	Low risk
Geremia 2006 [16]	-	-	-	-	+	+	+	Moderate risk
Gordon 2009 [72]	+	+	-	-	+	+	+	Low risk
Gomez-Pinilla 2002 [13]	+	+	-	-	+	+	+	Low risk
Herbison 1973 [43]	-	-	-	-	+	+	+	Moderate risk
Herbison 1974 [44]	-	-	-	-	+	+	+	Moderate risk
Herbison 1980 [45]	-	-	-	-	+	+	+	Moderate risk
Herbison 1986 [46]	-	-	-	-	+	+	+	Moderate risk
Hines 1942 [47]	-	-	-	-	+	+	+	Moderate risk
Huang 2012 [17]	+	+	+	+	+	+	+	Low risk
Ilha 2008 [48]	+	+	-	-	+	+	+	Low risk
Inoue 2011 [73]	-	-	-	-	+	+	+	Moderate risk
Irintchev 1990 [49]	-	-	-	-	+	+	+	Moderate risk
Irintchev 1991 [50]	-	-	-	-	+	+	+	Moderate risk
Jaweed 1982 [51]	-	-	-	-	+	+	+	Moderate risk
Kao 2013 [20]	+	+	-	-	+	+	+	Low risk
Kim 1998 [52]	+	+	-	-	+	+	+	Low risk
Kluding 2012 [74]	+	+	+	+	+	+	+	Low risk
Lange-Maia 2016 [75]	-	-	-	-	+	+	+	Moderate risk
Liao 2017 [19]	+	+	-	-	-	+	+	Moderate risk
López-Álvarez 2015 [53]	+	+	+	+	+	+	+	Low risk
Love 2002 [54]	-	-	-	-	-	+	+	High risk
Marqueste 2003 [6]	+	+	-	-	+	+	+	Moderate risk
Marqueste 2006 [55]	+	+	-	-	+	+	+	Moderate risk
Martins 2011 [56]	+	+	+	+	+	+	+	Low risk
Martins 2017 [57]	-	-	+	+	+	+	+	Low risk
Mennen 2002 [76]	-	-	-	-	+	+	+	Moderate risk
Michel 1989 [58]	-	-	-	-	+	+	+	Moderate risk
Molteni 2004 [7]	-	-	+	+	-	+	+	Moderate risk
Pachter 1989 [8]	-	-	-	-	+	+	+	Moderate risk
Piccinini 2020 [77]	+	+	+	+	+	+	+	Low risk
Sabatier 2008 [12]	-	-	-	-	+	+	+	Moderate risk
Sarikcioglu 2001 [59]	-	-	-	-	+	+	+	Moderate risk
Seburn 1996 [60]	+	+	-	-	+	+	+	Low risk
Seo 2006 [61]	+	+	+	+	+	+	+	Low risk
Seo 2009 [21]	+	+	+	+	+	+	+	Low risk
Sinis 2008 [62]	+	+	+	+	+	+	+	Low risk
Skouras 2009 [63]	-	-	-	-	+	+	+	Moderate risk
Sobral 2008 [64]	-	-	-	-	+	+	+	Moderate risk
Soucy 2013 [65]	+	+	+	-	+	+	+	Moderate risk
Tam 2001 [66]	+	+	+	+	+	+	+	Low risk
Teodori 2011 [67]	+	+	-	-	+	+	+	Low risk
Udina 2010 [68]	-	-	-	-	+	+	+	Moderate risk
Van Meeteren-Wiegant 1997 [69]	+	+	+	+	+	+	+	Low risk
van Meeteren–Gispen1997 [70]	+	+	+	+	+	+	+	Low risk
Werning 1991 [71]	-	-	-	-	+	+	+	Moderate risk
Wong 2015 [78]	+	+	+	+	+	+	+	Low risk

+ indicates reporting in full with low risk of bias; - indicates no reporting with high risk of bias.

## 3. Results

### 3.1. Description of the Studies

**Figure 1:**
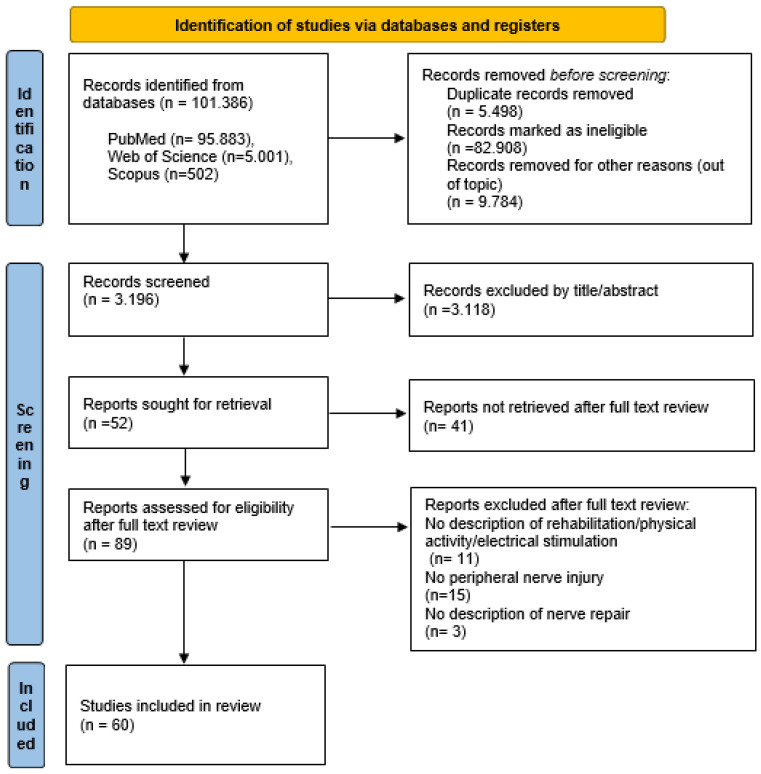
Flow chart of the process of initial literature search and extraction of studies meeting the inclusion criteria.

The literature search identified 101,386 articles. The authors reviewed the titles and abstracts, and 89 papers were selected for full text screening; suitability of inclusion in the study was independently assessed. Sixty publications met the inclusion criteria and were included in the systematic review. At the end of the search process, 29 articles were excluded for the following reasons: 15 did not consider peripheral nerve injuries, 11 did not describe any physical exercise, and 3 did not describe the nerve repair process. The number of studies produced at each stage of the search is shown in Figure 1. The sample characteristics and the design details of each study are shown in Table 1 for animals and Table 2 for humans. The evaluation of the quality of the studies is presented in Table 3, the assessment of risk of bias is shown in Table 4.

### 3.2. Variations of Study Characteristics across the Studies and Risk of Bias

As reported in Table 1 and Table 2, most of the studies were on animals, especially rats, and 7 articles regarded human participants.

The paucity of significant positive results made an evaluation for humans incomplete and uncertain; however, the study on humans is worthy of further investigation as a subject that is still little debated and for which it is necessary to stimulate more interest.

There was a large variation among the studies concerning the proposed activity, regarding the type, duration, and start of intervention from nerve repair, assessed parameters and evaluations.

To reduce the risk of bias, the results were considered separately for humans and animals. Nevertheless, most preclinical studies used different animals, in number, type, age, and weight. Moreover, the included studies used different treatments, regarding timing, duration, type of exercises, and intensity of electrical stimulation. Because of the heterogeneity of the therapeutic strategies, a quantitative analysis of the results from the different interventions and the start of treatment to understand the best strategy was not possible.

### 3.3. Outcome Measurements

Electrophysiological parameters, Semmes–Weinstein monofilaments, contractions, and characteristics of muscle (i.e., weight), SFI, skin, nerve or muscle biopsy were used to evaluate nerve damage and repair processes.

In humans, electromyography (EMG) was used to reveal nerve lesions [73,76,77]. The electrophysiological recordings of nerve conduction studies focused on amplitude, latency [75], and unit number estimation (MUNE) [72]. The use of Semmes–Weinstein monofilaments showed the reinnervation of receptors that confer tactile function [78]. A proximal skin biopsy showed intraepidermal nerve fiber branching [74].

In animal research, nerve conduction studies were also used [6,11,19,31,40,52], especially for the analysis of nerve conduction velocity [37,70] and compound muscle action potentials (CMAPs) [17,20,43,68]. Nociceptive withdrawal thresholds [34,79] and thermal and mechanical thresholds [53] were also documented. Furthermore, muscles reacted to physical exercise after the nerve repair process with an increase in weight [19,42,43,46,47,49,50,51,55,58] and fiber diameter [38,43,44,52,64,67]. Tetanic tension was used to assess nerve repair [37,41,42,46,49,50,58,60] as well as isometric contraction [8,37,45,46,47,50,51]. Additionally, the analysis of SFI was another interesting parameter used [17,48,56,57,61,64,67,69,70]. Furthermore, histological nerve examination [32,33,34,35,36,38,44,65,71], in particular immunohisto-fluorescence [7,39,54,61] and immunohistochemistry [8,12,16,59,66], were diagnostic parameters. Likewise, the levels of neurotrophins and neurotropic factors were considered as parameters of nerve repair [7,12,53], in particular the expression of BDNF, its signal transduction receptor (trkB), and molecules associated with its action [7,10,13,14,34,39,53,65,79,80], the levels of GAP-43 mRNA [21,62], NGF [79], and hosphor-ERK1/2 protein [21]. Lastly, functional recovery was assessed by video-based motion analysis that precisely evaluated muscle function during locomotion [30,63]. This complexity of mechanisms involved in axon regeneration is shown in Table 5.

### 3.4. Electrical Stimulation and Physical Exercise: Intervention Characteristics and Effects on Peripheral Neuropathy

Electrostimulation of denervated muscle and locomotion exercises were shown to be effective strategies in retarding muscle atrophy and improving contractile response after reinnervation [6].

Most of the animal studies used a low-frequency electrical stimulation at 20 Hz [11,16,17,30,33,39,54,63,66] or at 10 Hz [20,35,46,51], other studies used a variable frequency, at 0, 2, 20, or 200 Hz [20], at 100 or 150 Hz [32], at 20 or 100 Hz [54], and from 4 Hz (200 ls) to 75 Hz [6,55].

In humans, functional electrical stimulation at 20 Hz was proposed by two authors [72,78], and at 1 Hz by only one author [77].

In regard to the kind of proposed exercise, most of the animal studies used the treadmill [12,31,37,41,45,57,61,68,71], high speed exercise (running) [34,49,50,53,60,64,66], swimming [19,36,44,59,67], voluntary locomotor activity [7,13,42,65,69,70], overwork [43,47,58], endurance and resistance exercises [48], isometric exercises [8], sensory retraining [40], manual stimulation [62], continuous passive motion [52], joint mobilization [56], and constraint therapy [38].

In humans, rehabilitation was proposed by four authors, with self-guided exercises [73], aerobic and strengthening exercises and task-oriented activities [74,75], sensory re-training and motor contraction exercises [76], with few positive results.

In animals, a total of nine studies measured the role of exercise or electrical implications applied early in the process of nerve repair, that is, immediately after nerve injuries, until 3 weeks after denervation, and before the period of reinnervation or during the early stage of reinnervation [44]. These studies supported both positive effects after electrical stimulation [17,20] and physical exercise [43,67,68]; however, there were also negative effects after exercise [66], especially when too intense [44], with a high variability of results [48,64]. Conversely, a total of six studies measured the effects of exercise or electrical stimulation applied during late stage reinnervation, that is, 4 weeks after denervation [44]. In the late stage, only one study documented positive effects after electrical stimulation [17]. Regarding physical exercise, a total of four studies reported positive effects [67,69,70], and two reported negative [48] and no effects [64] at late stage reinnervation. In regard to these articles, a total of six were of high quality according to SYRCLE’s risk of bias tool [17,20,48,67,69,70]. No data were presented about exercising early after nerve repair in humans.

Concerning duration, Cobianchi et al. [34] considered short-lasting exercises, when they lasted 1 h a day for not more than 5 days, and long-lasting training of 1 h a day for more than 5 days. On the one hand, short-lasting running had beneficial rehabilitative effects on functional recovery after peripheral nerve injury [34]. On the other hand, a prolonged electrical stimulation and long-lasting exercise had detrimental effects on peripheral nerve regeneration and neuropathic pain [11]. Of these articles, it is important to consider that one was of high quality according to SYRCLE’s risk of bias tool [34]. No data were presented about the timing of interventions on humans.

In conclusion, regarding the physical exercise described above, several types have been proposed in experimental nerve injury models; however, there have been conflicting results regarding their effectiveness on axonal regeneration. No standardized therapy has been applied, with differences especially in the choice of intensity, duration, and timing of programs. Despite this heterogenicity, this research showed that the literature agrees in affirming that especially early exercise programs promoted an axonal regenerative response and prevented maladaptive responses in animals.

Even if no studies in humans showed a clear relationship between rehabilitation and axon regeneration, certainly specific physical exercise avoids secondary injuries, such as contractures, disuse atrophy, stasis edema, and pain.

### 3.5. Voluntary, Forced, and Passive Exercises

Regarding the kind of exercise, the literature proposes voluntary, forced, and passive exercises.

In animal models, forced (an external source guided the exercise) and passive range-of-motion exercises had a standardized load of training on the basis of the study protocol, while for physical exercise, based on voluntary activities of animals, the research studied the response to the type of exercise. Passive exercise of the denervated muscle before reinnervation preserved a healthy structure and enhanced regeneration and reinnervation [8,52,68]. Combined rehabilitation of motor and sensory functions by passive and active physical exercise improved the coordination of sensory–motor tasks and restored adequate circuitry at the spinal level [68]. Moreover, both passive and active exercises promoted the regeneration of axons of distal nerves, as well as the reduction in the hyper-excitability of spinal reflexes after nerve injury [68]. However, only one article was of high quality according to SYRCLE’s risk of bias tool [52].

In humans, the proposed exercises included stretching to warm up, aerobic or strengthening exercises, cardiovascular training with body recumbent steppers, upright cycle, recumbent cycle, and treadmill training. Strength training included abdominal curls, biceps curls, chest presses, lat pulldowns, leg extensions, seated leg curls, seated rows, shoulder presses, squats, and triceps presses [74], sensory re-training and motor contraction exercises [76]. Suggested intense activities included walking, jogging, swimming, singles tennis, or moderate activities included golf without a cart, doubles tennis or light activities (e.g., golf with a cart, shuffleboard), muscle strengthening exercises, lawn work and gardening, occupational activities that include walking or standing, caring for another person, home repairs, and housework [75]. Only one article proposed self-guided rehabilitation, with exercises that were carried out at home [73].

All these studies on humans described positive effects regarding functional recovery, improvement of symptoms and neurophysiological parameters.

### 3.6. Intensity of Physical Exercise and Recovery of Nerve Function

Concerning intensity, in animals, exercise was considered high when it lasted 2 h a day [44], moderate at 20 min/3 times/week [19], and low intensity was considered as a very undemanding load (i.e., 8 m/min of treadmill) [21].

Low-intensity physical exercise was described as positive in nerve regeneration [61]; indeed, it potentiated Schwann cell proliferation in regenerating the sciatic nerve in rats [61]. Sabatier et al. [12] showed that low-volume intermittent exercise in very small quantities, instead of continuous exercise at higher volume, enhanced axonal outgrowth.

Moreover, positive effects of nerve repair were also attributed to moderate intensity physical exercise [5,11,12,19,36], enhancing the regeneration of injured axons with differences related to sex [5,11].

Ten studies evaluated high-intensity exercise [34,49,50,53,60,64,66] and overwork activities [43,47,58]. Some described positive effects, except five articles that described negative effects [44,58,66], and two articles that described no effects [49,64].

Furthermore, the progressive increase in training intensity was described as positive in nerve regeneration processes by five articles [12,34,37,53].

Concerning the regeneration process, while continuous moderate or intermittent high-intensity physical exercise was able to promote axonal elongation after allograft repair, even if without increasing the sprouting index [12], a progressive increase in volume and/or intensity of exercise was an important factor in determining the rate of motoneuron sprouting [37] and was associated with neurotrophin upregulation [12]. Moreover, an early and progressive increase in training intensity reduced neuropathic pain [34,79] and prevented neurotrophin-mediated hyperexcitability of peripheral injured nerves [53] and hyperreflexia, as shown by the reduction in the facilitation of the monosynaptic H reflex [11] and the early reappearance of the H reflex with increased amplitude after exercise [31]. On the other hand, it was hypothesized that due to a feedback mechanism, large doses of BDNF inhibited axon regeneration [81]. Sprouting was inhibited in vivo by increased neuromuscular activity associated with wheel running in rats [66]. In fact, some evidence showed that muscle stimulation dramatically reduced the terminal sprouting that normally occurred following partial denervation [32,54]. Regarding increased daily neuromuscular activity preceding partial denervation, this did not lead to motoneuron sprouting responses of slow muscle, such as the soleus, but enhanced short-term sprouting of fast muscle motoneurons, such as the plantaris muscle [41]. This contrasted with the absence of enhancement of sprouting in rats subjected to daily exercises following partial denervation of the plantaris muscle [42].

Intense and prolonged physical exercise reduced the mechanical hyperalgesia of the sciatic nerve after its regeneration [53]. On the other hand, as reported, manual stimulation for injuries of mixed nerves [62] and electrical stimulation [63], especially if prolonged [11], did not improve functional outcomes. A prolonged electrical stimulation or an intense locomotion exercise had detrimental effects on peripheral nerve regeneration and neuropathic pain [11,34]. However, six articles were of high quality according to SYRCLE’s risk of bias tool [34,42,53,60,61,66].

Shared results of a progressive and high level of physical exercise were unclear.

In humans, concerning the load of exercise, the improvement in peripheral neuropathy was modestly associated with daily vigorous physical exercise [75].

## 4. Discussion

### 4.1. Summary of Collected Data

To our knowledge, this is the first systematic review looking at the role of activity-based rehabilitation and electrical stimulation in the processes of spontaneous or surgical nerve repair secondary to peripheral neuropathies. The effectiveness of activity-based training, rehabilitation and electrical stimulation on peripheral nerve regeneration and muscle reinnervation is controversial. The lack of guidelines regarding the type of exercises, duration, and intensity makes the results heterogeneous and not easily comparable. Furthermore, in most studies, the involved processes of nerve repair are not the principal focus.

This review presents important clinical considerations: the effectiveness of different physical exercises and electrical stimulations to improve the recovery of peripheral nerve injury, the possibility of application in humans and the detrimental effects of exercise too early after nerve injury. Indeed, it proposes promising patterns for further investigation, comparing (1) the outcomes and kind of rehabilitation proposed early and late in the process of nerve repair, (2) short- and long-lasting exercises, (3) different intensity (from high to low), and (4) progressive versus sudden high load.

### 4.2. When to Start Treatment

The effectiveness of physical exercise could be related to the time of the start of activity. In fact, it has been shown that starting exercises for denervated muscles early, immediately after injury, contributes to accelerating nerve regeneration and synaptic elimination [67]. In particular, early progressive resistance exercises in the presence of axonal dysfunction could enhance spontaneous neurologic recovery [82], and physical exercise during the denervation period could cause more positive effects compared to performing exercises during reinnervation and the recovery period [49]. Moreover, treadmill training during the early and late phases of nerve regeneration after crushing the sciatic nerve of rats did not influence axonal budding, degree of maturation of regenerated fibers or the functionality of the reinnervated muscles [64]. However, according to a few authors, exercise seems to be effective starting from the 4th regeneration week after nerve injury and not before [59].

According to other authors, increased neuromuscular activity was not recommended immediately after motoneuron injury or in the early stages of motoneuron disease [66]. Additionally, high workload, over-training and overuse proved to interfere with peripheral nerve recovery, especially in the early phase of recovery [44]. Indeed, an excessively early start of treatment (before the period of reinnervation) and intense physical exercise had detrimental effects [44]. On the contrary, long-lasting [34], high-intensity training, overload, and overwork, especially at the onset of reinnervation [43], or increased neuromuscular activity in extensively denervated muscles [66] represented an unphysiological stimulus that interfered with normal anatomical and biochemical recovery [44,66].

### 4.3. Specific Physical Programs and Conflicting Views in the Literature

Regarding the kind of proposed exercise, passive and active exercise is effective in nerve repair processes [8,52,56,68], including voluntary exercise [7]. Combined therapies for motor and sensory recovery with passive or active exercise programs could improve the coordination of sensory–motor tasks and restore an adequate circuitry at the spinal level [68].

Specific exercises such as eccentric [57], endurance [48], and sensory rehabilitation [40] are described as effective in nerve regeneration processes. In particular, regular eccentric exercise improves morphological nerve regeneration, reduces mechanical and cold hyperalgesia, and accelerates motor functional recovery [57]. Sensory rehabilitation [40,76], especially with intensive programs of sensory rehabilitation after regeneration, can be used to improve even sensory perception [40].

On the contrary, resistance exercises and immobilization seem to delay functional recovery [38,47,48]. Too many heterogeneous results were recorded for manual stimulation [62,63]. In particular, resistance exercise or the combination of resistance and endurance training may delay functional recovery, but do not alter sciatic nerve regeneration, while moderate endurance exercise improves nerve regeneration [48].

Immobilization seems to have no detrimental effects on the count of number of fibers from a limb compared to contralateral active limb [38], but it delays recovery, probably because of a reduction in muscle regeneration rate rather than because of an influence on nerve regeneration [47].

### 4.4. Electrical Stimulation and Conflicting Views in the Literature

Concerning electrical stimulation, positive effects in the nerve repair process were recorded in both animal [6,16,20,33,35,39,46,55] and human research [72,78]. The findings indicate that it is efficient for maintaining muscle weight, but other parameters, such as twitch characteristics, fatigue index, mechanosensitivity, and metabosensitivity, are totally restored when an animal performs a running exercise during the rehabilitation period [6,55]. High-frequency electrical stimulation has been shown to exert benefits in diabetic peripheral neuropathies [20]. Brief electrical stimulation leads to accelerated locomotor recovery [30] and facilitates reinnervation [33,72,78]. The chronic and prolonged stimulation of intact axons of partially denervated muscles enhances muscle recovery [6,55] and increases muscle weight and the tension of the electrically stimulated muscle [46]. Moreover, electrical stimulation of the facial nerve did not improve the functional outcome nor reduce aberrant regeneration after facial nerve reconstruction in rats [63].

The only positive effect of this treatment on the facial nerve was a transient improvement of protraction velocity between 1 and 3 months after surgical reconstruction [63].

Acute electrical stimulation appears to exert some transient benefit in small animal laboratory models following injury to mixed peripheral nerves; thus, this intervention does not confer any long-term benefit following injury to a purely motor nerve.

In humans, clinical use of direct current electrical stimulation of acupuncture needles together with self-guided rehabilitation for motor function recovery after neurapraxia and axonotmesis was suggested for peripheral nerve damage with positive results [73].

### 4.5. Electrophysiological Parameters, Muscle Characteristics, and Neurotrophic Mechanisms

The electrophysiological changes related to the start of treatment, secondary to physical exercise and direct stimulation, included CMAP latency and amplitude both in rats and in humans, nerve conduction velocity in humans, SFI, muscle fiber and axon diameter in animals to highlight the differences, the possibility of recovery or detrimental effects related to rehabilitation of nerve repair processes after injury.

In humans, when EMG reveals complete nerve lesions, the reinnervation is generally unlikely to happen after electrical stimulation [77]. Furthermore, it is important to highlight that it is possible that the improvement of the parameters was partially explained by the natural history of the disease [77]. In particular, amplitude and latency are indicators of different types of peripheral nerve damage, with worse amplitude indicative of axonal degeneration, while latency, a component of conduction velocity, is a sign of demyelination [75]. Nevertheless, in the early stage, an absent EMG response did not prove failure of nerve growth or re-innervation due to smaller axons, which need to be myelinated and that lasted for a long time (up to three years) [76]. Axonal regeneration is quantified using motor unit number estimation (MUNE) and sensory and motor nerve conduction studies [72]. Semmes–Weinstein monofilaments show reinnervation of receptors that confer tactile function [78]. A proximal skin biopsy shows intraepidermal nerve fiber branching [74].

In regard to the start of treatment, the studies on electrophysiological changes described encouraging results when rehabilitation was proposed early. Indeed, when therapeutic interventions started early (immediately or within the first weeks after nerve injury), the literature revealed changes in electrophysiological recordings of CMAPs for latency.

When therapeutic strategies started early, the SFI in rats showed increased values [64,67] until the first week after nerve injury, while decreased values were observed until the second and the third weeks, indicating significant functional loss [48,64,67]. Highly variable results were recorded when the start of treatment was not until the fourth week, reporting both decreased [17,48] and increased values [64,67,69,70]. Physical exercise significantly improved the value of SFI when it started early, within 2–3 weeks from nerve injury.

In animals, muscles reacted to physical exercise with an increase in weight, as well described for the plantaris [41,42,58] and the soleus muscle [49]. On the other hand, no effect of training was shown in muscles atrophied after denervation [37,44]. Stimulation for a brief period (three minutes a day) greatly retarded muscle atrophy prior to reinnervation and accelerated the recovery of muscle weight and strength subsequent to reinnervation. This was documented for gastrocnemius muscles [47] and chronic stimulation (for six to ten weeks) of intact axons of partially denervated muscle increased muscle weight, as documented for the soleus muscle [46,55]. Furthermore, a considerable increase in diameter of myelinated axons after electrical stimulation was shown [17,64,67].

The regenerative mechanisms were connected to the production of neurotrophic factors. Collateral sprouting and axonal elongation of undamaged axons occurred in response to denervation due to local production of neurotrophic and neurotropic factors [53]. The levels of neurotrophins were related to physical exercise; they enhance axon elongation [12]. Regarding the type of training, voluntary exercise, even brief [7], increased axonal regeneration through a neurotrophin-dependent mechanism and neurite outgrowth [7,13,80].

### 4.6. The Rehabilitative Point of View and Implications for Humans

Too many differences exist between animals and humans, i.e., genetics, habits, adherence to treatment, and comorbidities that could influence outcomes. For example, exercise could modify the natural history of diabetic peripheral neuropathy; for this reason, human studies must be encouraged. Indeed, in people with diabetic peripheral neuropathy, a short-term intervention with a supervised aerobic and resistance exercise program seems to exert beneficial effects on pain and neuropathic symptoms [72].

At the same time, a study by Lange-Maia et al. [75] showed that in elderly humans, a reduction in discomfort related to peripheral neuropathies was associated with higher levels of self-reported physical exercise and more daily minutes of objectively measured vigorous activity. In an interesting report, at peripheral nerve testing, sensory amplitude (SNAP), indicative of axonal degeneration, was associated with objective vigorous physical exercise [75], while distal motor latency, a component of conduction velocity related to demyelination, was associated with self-reported physical exercise [75]. Thus, a relationship between a better motor nerve function and higher levels of physical exercise was proposed [75]. Moreover, the extent of the damage dictates the need to proceed with appropriate strengthening programs [82].

The timeliness, the choice of the type of rehabilitative exercises, and symptomatology are essential for successful treatment. Moreover, there is evidence that the recovery of sensory and motor nerves is not identical. Furthermore, individual differences in recovery of function after peripheral nerve damage could be related to the origin of the peripheral neuropathy, pre-existing biological traits, hormonal balance, and behavioral characteristics [69]. For this reason, rehabilitative programs should be personalized.

## 5. Limitations

Differences in the kind and number of animals with respect to humans, as well as the types and duration of interventions, may affect the results of the present review. Different typologies of animals, mostly rats of different species, but also rabbits and snails result in the samples being very heterogeneous. Furthermore, human subjects vary in age and their comorbidities are not always sufficiently described. Moreover, few studies examined the voluntary activities of animals, thus a standardized control of the intensity, duration, and pattern of training is missing compared to organized and controlled passive or active activity programs. Indeed, when animals are free to move, their exercises are not comparable to a measurable and preventive chosen training. Furthermore, the type of exercise used in the various studies is very different. Indeed, active, passive, or controlled exercises are proposed, with different intensity (high, moderate, or low) as well as duration and timing (early or late starting). A similar heterogeneity is present for electrical stimulation: it is mostly used at 20 Hz, but the frequency is also variable in some articles. This heterogeneity did not allow a quantitative analysis of data, but it suggests new promising research to understand the ways in which exercise and electrostimulation can influence nerve repair.

## 6. Conclusions

In most preclinical studies, peripheral neuropathy function was associated with improvements due to physical exercise and electrical stimulation. For humans, limited research has been conducted on this topic to reach a complete evaluation. Certainly, it would be desirable to determine an effective therapy for peripheral neuropathy in humans. Unfortunately, physical exercise often has no effects on the course of peripheral neuropathies; in fact, in rats, both detrimental and beneficial effects have been described. Thus, this study shows that the literature is inclined to propose the thesis that an early physical program with active exercise and/or electrical stimulation promotes an axonal regenerative response and prevents maladaptive responses, with changes in electrophysiological recordings.

The degree to which the types of activity or the duration of exercise could influence outcomes is unclear. In humans, only a few studies with patients with diabetes indicate that exercise could reduce the symptoms related to neuropathies; however, robust trials are necessary to understand whether controlled physical exercise and consequent specific rehabilitation plays a role in regeneration and repair of injured nerves and denervated muscle.

This study intends to spur new research to explore the possible mechanisms of activity-based rehabilitation on peripheral neuropathies, as well as immediate and long-term effects. Moreover, our findings support the need for future research to test the validity of a possible rehabilitation treatment in cases of peripheral neuropathy to help maintain functional independence.

## Figures and Tables

**Table 5:** Mechanisms of action of controlled physical exercise on nerve repair and regeneration.

Kind of Exercise	Action of Exercise on Nerve Repair	In-Depth Features	References
Modulator of the Neurotrophins			
Physical exercise	Increment of neurotrophin levels	For regenerating neurons	[7,14,34,79]
	Increment of neurotrophin levels such as BDNF	For the survival and regeneration of injured axons	[7,14,39]
	Induction of GMF and BDNF	GMF could be necessary for exercise induction of BDNF and could promote neuroprotection through BDNF production	[18]
High-intensity physical exercise	Reduction both in early hyperalgesia, decreasing the production of NGF in the skin and in sensory neurons, and late hyperalgesia related to reinnervation by regenerating nerve fibers	Early hyperalgesia is associated with collateral sprouting of intact nerve fibers	[79]
High-intensity physical exercise	Reduction in BDNF at the level of microglia and dorsal root ganglia	Modulation of neurotrophin mechanisms that regulate growth and excitability of sensory neurons after peripheral nerve injury	[53]
Treadmill running	Hyperalgesic responses are strongly dependent on NGF	The early reduction in hyperalgesia is likely associated with the reduction in local NGF production	[79]
Low intensity, but not high intensity	Low-intensity, but not high-intensity treadmill increased neurite outgrowth of dorsal root ganglion (DRG) sensory neurons and potentiated Schwann cell proliferation	Treadmill elevated levels of GAP-43 mRNA and protein, and hosphor-ERK1/2 protein in the injured sciatic nerves	[21]
Voluntary exercise	Increase in axonal regeneration through a neurotrophin-dependent mechanism and neurite outgrowth	Increase in expression of several molecules associated with the action of BDNF on synaptic function	[13,65,80]
Voluntary exercise	Sensory ganglia from the 3- and 7-day-exercised animals contained higher brain-derived neurotrophic factor, neurotrophin 3, synapsin I, and GAP43 mRNA levels than those from sedentary animals	Increase in axonal regeneration after 3–7 days of exercises through a neurotrophin-dependent mechanism	[7]
Voluntary exercise	Modulation of neurotrophin signal	Regulating the growth of sensory neurons	[7]
Regular eccentric exercise	Reduction in TNF-α in the muscle and increase in IGF-1 in nerve. Activation of serotoninergic and noradrenergic systems (descending pain inhibitory systems) improved morphological nerve regeneration	In sciatic nerve crush-subjected animals: reduced mechanical and cold hyperalgesia accelerated motor functional recovery	[57]
Electrical stimulation and exercises	Increase in BDNF and trkB expression	Increase in the expression of BDNF and trkB mRNA in regenerating femoral motoneurons	[10]
Brief electrical stimulation	Decrease in dorsal root ganglion neurons regenerating into cutaneous and muscle branches, increase in numbers of neurons that regenerated axons, and the expression of GAP-43 mRNA in the regenerating neurons and of BDNF	-	[16]
Electrical stimulation	Up-regulation of S-100, BDNF, Par-3	-	[17]
Swimming exercises	Increase nerve repair-associated makers, and calcitonin gene-related peptide (CGRP)	-	[19]
Sprouting			
High intensity	Inhibition of denervation and induction of early collateral sprouting	Hampering of longer duration nerve regeneration	[79]
Increase in axonal outgrowth			
Prolonged treadmill exercise	Promotion of enlargement of fast-fatigable and fast–intermediate motor units	At the level of partially denervated gastrocnemius	[37]
Electrical stimulation	FES-induced acceleration of axon regeneration in post-surgical carpal tunnel syndrome	Improved MUNE, motor units, terminal motor latency, sensory nerve conduction values	[72]
Regulation of neuronal cotransporters			
High-intensity exercises	Prevention of NKCC1/KCC2 deregulation	It is a nerve injury-dependent mechanism of central disinhibition	[53]

Growth-Associated Protein 43, GAP-43; Brain-Derived Neurotrophic Factor, BDNF; Nerve Growth Factor, NGF; Na-K-2Cl Cotransporter isoform 1, NKCC1; K-Cl Cotransporter isoform 2, KCC2; Motor unit number estimation, MUNE.
